# What Every Dentist Should Know

**Published:** 1875-07

**Authors:** G. B. Harriman


### WHAT EVERY DENTIST SHOULD KNOW.

BY G. B. HARRIMAN, D. D. S., M. D.

*Read at the Seventh Annual Meeting of the District Dental Society of the Seventh Judicial District of the State of New York, June 1 and 2, 1875.*

Every Dentist should know the different kinds of food necessary to form the different tissues which compose the human system. The food that goes to make and build up the muscular portion; that which is to make bone, teeth and brains; and that which gives heat to the system. At the present time nearly every department of labor has its colleges; the lawyer, the theologue, the physicist, the chemist, the dentist, and last but not least we have our agricultural colleges to teach the science of cultivating the earth. If farming is an important science in demonstrating by analysis, the soil, the best vegetables and grains to be grown upon it, and the different manures necessary to supply whatever elements are important and lacking to make the grain grow in the most profitable manner, can we as dentists, engaged in a nobler and more scientific calling which combines the most artistic skill in manipulating taste and intellectual thought to produce the best results, be satisfied, while we don't know the elements that we are operating upon, and the food necessary to build each and every part of the human fabric, to make it sound and durable? The composition of the human body consists of fourteen different elements as follows: Oxygen, Hydrogen, Nitrogen, Carbon, Phosphorus, Calcium, Fluorine, Chlorine, Sodium, Iron, Potassium, Magnesium, Silicon and Sulphur, all of which are found in food grown on cultivated soil; and the human body can not exist a single day without the waste of some portion of these elements. They must be supplied in the atmosphere, water and food. The food we will divide into three classes, nitrogenous, carbonaceous and phosphatic. The nitrogenous is that part which supplies the muscles; The Carbonaceous is the portion which supplies the lungs with fuel and furnishes heat to the system, called *fat;* the phosphatic elements supply the teeth, bone and brain, and give vital action to the whole body, The waste and supply of these elements is very diverse; two and one-half per cent of the phosphatic, twenty per cent of nitrogenous, and seventy per cent or a little over of carbonaceous, are about the average proportionnecessary with moderate exercise of the whole system. Where are these elements found in the right proportions? In wheat we find water, 14 per cent; nitrogenous or muscle making material, 14 per cent; cabonosseous, fat or starch for heat, 10 per cent; phosphate for osseous structures and nerves, two and one-half per cent. These three principles are made up of the 14 elements which compose the human body; and the proportions of muscle making, heat producing, bone and brain feeding elements are about the usual ratio required in moderate weather, and exercise of the mental and physical faculties. The scientific farmer knows that wheat will not grow in soil out of which has been taken any of the essential elements that constitutes that grain; and he either supplies these elements or does not try to raise it.

Yet how may dentists there are in the profession who do not know the elements of the human body, their chemical or physiological structure, and hence are ignorant of the proper hygienic treatment to advise in cases where it would be of great value. The soft and chalky teeth of children and young people can be greatly improved by care, exercise and *diet.* We will name some of the various sources of nourishment most essential to produce good results, oat meal is rich in nitrogenous and phosphatic substances, which go to produce muscle, teeth and brain. Unbolted wheat flour will produce good results. Beans and peas are exceedingly nutritious; they contain a very large per cent of nitrogenous (muscle making) ingredients. Beef, mutton, venison, and trout, salmon, and many other kinds of fish are rich in nitrogenous and phosphatic substances. Cheese contains about 64 per cent of nitrogenous, 8 per cent of phosphatic, and 18 per cent of carbonaceous substances. Milk is very nutritious. The above diet in cases where the teeth have a decided tendency to crumble and disintegrate should, in my mind, be recommended in heroic doses. Superfine flour, pies, sweet cake, fats and starches of all kinds, in cases where the teeth have a tendency to decay badly, should be used sparingly, if at all, for they not only help the decay, but disorganize the whole fabric. Most persons, as every operator knows, who have soft decaying teeth, have a very delicate, nervous organization and small vital energy, more or less hereditary of disease, and suffer extreme pain when undergoing dental operations. "Can we do anything for patients so organized?" asked one dentist. I believe we can help them very much if they follow our advice; for it is a well known fact in the medical profession that the starch diet which prevails so generally brings many diseases such as nervous debility, feeble constitutions, lung complaints, heart disease, weak and defective eyes, early grey hair and many other ills, which carry so many young and middle aged people to a premature grave. This in a measure can be prevented—not wholly in our day perhaps—but we can commence this reform, and if the dental profession will work unitedly their efforts will produce immense good; and should it be continued by those who are to follow in our footsteps, the children of coming generations will be wonderfully benefited.

Prof. A. Lawrence, in his able report on Dental Chemistry, at Cincinnati, in 1867, before the American Dental Association, says, "What we are, results in some degree from what we eat and drink. Here permit me to suggest, with due deference, that this association ought, in justice to its professional behests, to deprecate the dictates of the fashion in dietetics which virtually starves us in the midst of our surfeits of fine, refined and superfine food. We sift from our cereals the best portions of the grain, and

"To eat the worse we most incline,
And feed the best to filthy swine."

The above remarks of the able brother should be read carefully for they contain much truth. Prof. Taft, in an editorial of the Dental Register of March, 1873, in speaking of the causes of dental caries, says, "As in part accounting for these defects, it is held that most civilized nations fail to take into the system a sufficient amount of tooth and bone forming material. The enamel and dentine are thus found under conditions which prevent a due degree of density and thorough calcification. The remedy for this condition in the child whose osseous tissues are still forming is found in the daily use of oat meal, barley, unbolted wheat flour, and such other vegetable and animal aliments as contain an abundant supply of the mineral salts. Many dental practitioners have sought to correct the deficiency of mineral salts in the blood, from which the teeth draw their nutriment by administering phosphate of lime, which is by far the most abundant mineral ingredient of the teeth. How far this treatment has been successful is not yet known; there has been no extensive record of cases. It is manifestly good practice to furnish the deficient substance as far as possible through wholesome foods that contain them. At least such practice can do no harm, and is probably a more natural mode of supplying them. Oysters, beefsteak, milk, beans, peas and barley as well as wheat, are rich in the mineral salts that are concerned in building up enamel and dentine, and should be abundantly supplied to all children. A deficency of mineral salts in the soil in which wheat and other grains are sown, will produce a feeble crop. It is the same with all vegetables, take the potato different observers state the percentage of magnesia in the ash of sound tubers at from four to five per cent, in diseased tubers an analysis shows less than two per cent. By analysis of several other kinds of vegetables it was found to be true between healthy and diseased structures. Prof. Troupe found a deficiency of lime and magnesia in diseased orange trees. As with the vegetable kingdom so with the animal.

Whenever a structure is arrested in its development and still continues to grow abnormally it becomes chaotic, and this seems to be the fact at the present time in regard to the teeth where people live on "Superfine Flour" where by analysis, we find little less than half of one per cent of phosphatic substances when a whole grain contains five times the above amount. E. Cutter, one of our most distinguished physicians, who has written more or less upon this subject says "the diminution of mineral varies from two-thirds to four-fifths. In other words by the use of flour mankind loses from $\frac{2}{3}$ to $\frac{4}{5}$ of the elements that go to make up teeth and structures. It seems to me that every dentist who has any desire or pride to conserve the natural teeth should look at this subject in its true light, and carefully digest it, that they may be able to give skilful directions and advice to all who may be willing to receive it. I have tried the "superfine" flour diet in my own family, until I was satisfied of its dangerous character, and have changed it for the unbolted flour with plenty of good beef and nutritious food; and the result I can assure all is exceedingly gratifying. First, eat no "superfine flour" and starches, and allow as little cooked in your family as possible. Second, eat unbolted wheat, oat meal, barley, beans and peas, good beef and mutton, trout, salmon and other nutritious aliments which contain the necessary amount of nitrogenous and phosphatic substances to make good teeth, bones, muscles, and brain, and recommend it to all patients whose teeth have marked tendency to decay.

